# Comparative Analysis of Colistin Resistance in Pseudomonas aeruginosa: VITEK® 2 Compact Versus Broth Microdilution Method

**DOI:** 10.7759/cureus.76646

**Published:** 2024-12-30

**Authors:** Kajal S Yadav, Satyajeet Pawar, Suvarna A Yadav, Satish Patil

**Affiliations:** 1 Microbiology, Krishna Institute of Medical Science, Krishna Vishwa Vidyapeeth, Karad, IND

**Keywords:** broth micro-dilution, colistin, minimum inhibitory concentration (mic), pseudomonas aeruginosa, vitek® 2 compact

## Abstract

Background

Colistin, a last-resort antibiotic, has witnessed a surge in resistance, posing a significant threat to public health. Accurate and timely detection of colistin resistance is crucial for effective clinical management. This study aims to compare two commonly used methods, the VITEK® 2 Compact (bioMerieux, Marcy-l'Étoile, France) system and broth microdilution (BMD), for identifying colistin resistance in clinical isolates.

Materials and methods

The study was carried out in the Microbiology Department at Krishna Institute of Medical Sciences, Karad. The laboratory processed the specimens for identification, followed by antimicrobial susceptibility testing using the automated VITEK® 2 Compact system and the gold-standard BMD method.

Results

A total of 92 *Pseudomonas aeruginosa *isolates were examined using automation (VITEK® 2 Compact) and the BMD methods, and the findings were analyzed. A gender-based analysis of the study population showed that 67% were males and 33% were females. Among the various specimens received, the highest number of colistin-resistant isolates was found in urine (38 isolates, 41.30%), then pus (25 isolates, 25.17%), endotracheal tube (13 isolates, 14.13%), sterile fluid (7 isolates, 7.60%), blood (3 isolates, 3.26%), sputum (3 isolates, 3.26%), vaginal swab (2 isolates, 2.17%), and catheter (1 isolate, 1.08%). Of the 92 *P. aeruginosa* isolates, 59 (64.1%) were resistant to colistin by the BMD, while 41 (44.56%) were resistant by the VITEK® 2 Compact.

Conclusion

Colistin is increasingly used for multidrug-resistant Gram-negative infections. In this study, some isolates showed differing results between VITEK® 2 Compact and BMD. The statistical analysis showed moderate kappa agreement, confirming the consistent reliability of the VITEK® 2 Compact system for testing colistin minimum inhibitory concentrations. Consequently, we can employ the VITEK® 2 Compact automated system as an alternative.

## Introduction

Multidrug-resistant (MDR) Gram-negative bacilli have emerged as a global health challenge. *Pseudomonas aeruginosa *is a well-known opportunistic pathogen commonly associated with infections in ICUs. This is a leading cause of acute pulmonary healthcare-associated infections and severe infections, particularly in immunocompromised patients [1]. Colistin is frequently a last-resort medication used for *P. aeruginosa* infections caused by MDR and extensive drug resistance strains, and detection of colistin resistance is essential for the management of infected patients. In order to treat infected patients, colistin resistance must be identified. Polymixin E, also referred to as colistin, was identified in 1949 and produced by the spore-forming soil microorganism *Paenibacillus polymyxa* subspecies *colistinus*. It is a component of the polymixin class of antibiotics, which also includes polymyxin A, B, C, and D. Only polymixin B and colistin E are used in human clinical settings. It exhibits superior bactericidal efficacy against a range of aerobic Gram-negative bacteria [2]. Colistin is an antimicrobial and polycationic medication that acts on lipid lipopolysaccharide moiety, displacing its cationic charges and causing microbial mortality and cell wall lysis [3]. Rising resistance to colistin results from its increased use, which is a clinically concerning development [3].

Despite decades of clinical application, the appropriate approach for polymyxin susceptibility testing has yet to be found. Furthermore, microbiologists must follow defined methods while testing sensitivity to ensure precise and correct results. However, with the emergence of MDR Gram-negative bacteria and the associated increase in colistin use, scientists had the chance to develop quick and precise means to test a colistin resistance isolate's sensitivity, and there is currently a critical demand in clinical laboratories [4]. Presently, testing for polymyxin sensitivity offers a significant concern because infections with Gram-negative bacteria resistant to colistin have been associated with increased patient mortality [5]. Many variables make testing for polymyxin difficult, including the compound's poor agar diffusion, cationic nature, the fact that many species exhibit hetero-resistance to the compound, and the lack of a reliable reference method that allows for accurate comparisons of commercial tests. Exact methods for antimicrobial susceptibility testing (AST) of colistin are vital, yet significant inconsistencies have been documented among the available tests. To tackle this problem, the Clinical and Laboratory Standards Institute (CLSI) and the European Committee on Antimicrobial Susceptibility Testing (EUCAST) have created the polymyxin initiative. Broth microdilution (BMD) was recommended by the Breakpoints Working Group for colistin susceptibility testing as the most valid method for colistin AST [6]. The integrated EUCAST/CLSI task force recently validated the issues with both of the available colistin gradient tests (made by bioMérieux and Liofilchem). For many years, all automated methods, such as VITEK® 2 Compact (bioMerieux, Marcy-l'Étoile, France) system and Phoenix system (BD, Franklin Lakes, New Jersey, USA), have been used to report colistin susceptibility results. CLSI guidelines 2018 published a modification as follows: the only acceptable minimal inhibitory concentration (MIC) method for testing is the BMD method [7]. So, this study aims to determine accurate MIC of colistin-resistant organisms and compare it with widely used methods, such as VITEK® 2 Compact.

## Materials and methods

This observational cross-sectional study was carried out in the Department of Microbiology at Krishna Institute of Medical Sciences, Karad. Specimens received in the laboratory were identified, and AST was conducted using the VITEK® 2 Compact with a Gram-negative card, while manual AST was performed using the gold-standard BMD method.

BMD

BMD was carried out according to the CLSI guidelines using a 96-well microtiter plate (HiMedia, Thane, India), colistin sulfate powder (Sigma Aldrich, St. Louis, MO USA), and cation-adjusted Mueller-Hinton broth (CAMHB). For the positive control, the MIC of mcr-1-positive *Escherichia coli* National Collection of Type Cultures (NCTC) 13846 ranged from 2 to 8 mg/L, and *P. aeruginosa* American Type Culture Collection (ATCC) 27853, used as the negative control, had an MIC ranging from 0.5 to 4 mg/L [8]. 

Colistin sulfate stock solution was first made using sterile distilled water, sealed in sterile plastic vials, and kept at -70°C until needed. In accordance with CLSI, a working stock solution was prepared from this stock solution by double dilution at concentrations ranging from 16 µg/mL to 0.5 µg/mL. The MIC was determined as the lowest concentration of colistin sulfate at which no visible growth occurred. This was performed to ensure the sterility, growth, and integrity of CAMHB and the ATCC strain. A colistin sulfate MIC of ≤ 2 µg/mL was regarded as the susceptibility threshold for *P. aeruginosa*, while a MIC of ≥ 4 µg/mL was considered the resistance threshold for *P. aeruginosa* [5].

VITEK® 2 Compact

VITEK® 2 Compact employs plastic reagent cards with microliter amounts of antibiotics and test media in wells. It examines colistin concentrations ranging from 16 μg/mL to 0.5 μg/mL and measures turbidometry to evaluate bacterial growth for a duration of 4 to 10 hours [9].

## Results

In the current investigation, all microorganisms were evaluated using the VITEK® 2 Compact and BMD procedures, and the results were compared to those obtained using BMD, which was recognized as the standard method.

This study observed a gender-wise study population of 69 (75%) males and 23 (25%) females.

**Figure 1:**
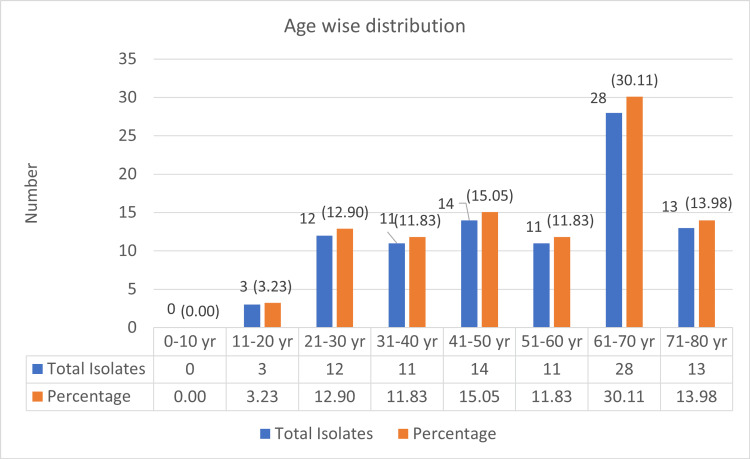
Age-wise distribution of infections with Pseudomonas aeruginosa isolates yr: Year

The highest prevalence of colistin-resistant *P. aeruginosa* isolates was observed in the 61-70 year age group, with 28 isolates (30.43%). This was followed by 14 isolates (15.21%) in the 41-50 year age group, 13 isolates(14.13%) in the 71-80 year age group, 12 isolates (13.04%) in the 21-30 year age group, 11 isolates (11.95%) in the 51-60 year age group, and 3 isolates (3.26%) in the 11-20 year age group (Figure 1)

**Figure 2:**
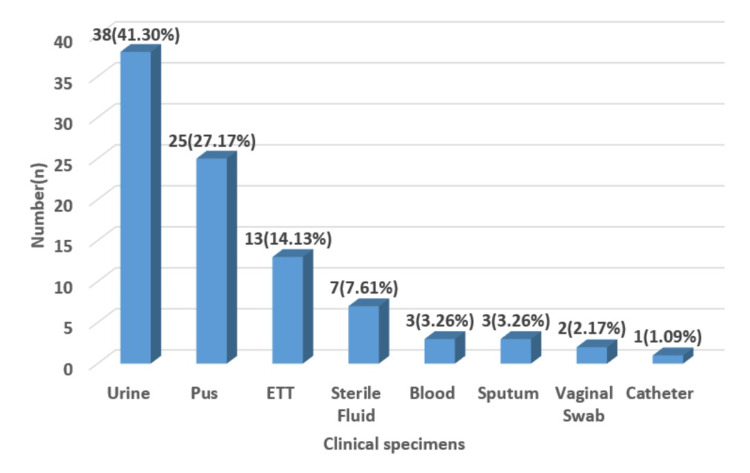
Specimens-wise distribution of Pseudomonas aeruginosa isolates ETT: Endotracheal tube.

Among the various specimens received, the highest number of colistin-resistant isolates was found in urine (38 isolates, 41.30%), then pus (25 isolates, 27.17%), endotracheal tube (ETT) (13 isolates, 14.13%), sterile fluid (7 isolates, 7.60%), blood (3 isolates, 3.26%), sputum (3 isolates, 3.26%), vaginal swab (2 isolates, 2.17%), and catheter (1 isolate, 1.08%) (Figure 2).

Comparison of VITEK® 2 Compact and BMD

A total of 92 *P. aeruginosa* isolates were analyzed using VITEK® 2 Compact and BMD methods, and the findings were analyzed for comparison. Among 92 isolates tested, 51 (55.43%) isolates were sensitive, and 41 (44.56%) were found resistant by VITEK® 2 Compact. In addition, 92 isolates of *P. aeruginosa* tested by BMD showed that 33 isolates (35.86%) were susceptible to colistin sulfate (MIC ≤ 0.5 µg/mL), while the remaining 59 isolates (64.13%) were resistant (Table 1).

**Table 1:** Comparison of colistin sensitivity test by VITEK® 2 Compact and broth microdilution methods

		Broth microdilution		
	-	Sensitive	Resistance	Total
	Sensitive	32	19	51
VITEK® 2 Compact	Resistance	1	40	41
	Total	33	59	92

The sensitivity of this test, as observed in this study, is 96.96%, and the specificity is 67.8%. The positive predictive value is 62.75%, while the negative predictive value is 97.56%. The correct prediction rate is 78.26%, and the expected agreement is 0.3173.

Kappa agreement statistics: 

$K = \frac{\text{Observed Agreement} - \text{Expected Agreement}}{1 - \text{Expected Agreement}}$ $K = \frac{0.7826 - 0.3173}{1 - 0.3173} = 0.6816$ Since k = 0.6816 lies between 0.6 and 0.79, it indicates that the agreement between the outcomes of susceptibility testing by the BMD and VITEK® 2 Compact methods is moderate.

MIC by BMD

A total of 59 out of 92 colistin-resistant isolates had an MIC ≥ 4 µg/mL by the BMD method, while 33 out of 92 colistin-sensitive isolates had an MIC ≤ 2 µg/mL by the same method [8]. Of the 92 *P. aeruginosa* isolates, 59 (64.13%) were resistant according to the BMD method (Table 2).

**Table 2:** MIC for colistin by broth microdilution method MIC: Minimum inhibitory concentration

No.	MIC (µg/mL)	No. of Isolate	Percentage of Isolate (%)
1	16	21	22.82
2	8	21	22.82
3	4	17	18.47
4	2	2	2.17
5	1	9	9.78
6	0.5	14	15.21
7	0.25	6	6.52
8	0.125	2	2.17
9	0.06	0	0.00
10	0.03	0	0.00

## Discussion

The spread of colistin-resistant Gram-negative organisms among ICU patients points to the challenge of improper antibiotic usage. As a result, a growing need for reliable susceptibility testing methods has arisen. In the current study, a comparison between automated and user-friendly VITEK® 2 Compact and BMD methods for colistin has been done in a clinical microbiology laboratory. A total number of 92 *P. aeruginosa* isolates routinely isolated from different clinical specimens were tested for colistin susceptibility by the BMD and VITEK® 2 methods.

In this study, 75% of the participants were male and 25% were female, which is consistent with the findings of a similar study where male predominance was significantly higher than female. Comparable results were reported from Telangana, where 62.5% were male and 37.5% were female [7].

In the current study, colistin-resistant organisms were most commonly observed in urine samples (41.30%), then pus (27.17%), ETT (14.13%), blood (3.26%), sterile fluid (7.60%), sputum (3.26%), vaginal swab (2.17%), and catheter (1.08%). This finding was comparable to Arjun et al., who also identified urine samples (33%) as the primary source of colistin-resistant isolates, followed by respiratory samples (20.8%), pus (16.67%), blood (6%), and cerebrospinal fluid (4.17%) [10]. Similarly, Pawar et al. reported colistin-resistant bacilli predominantly in pus (42.3%), then catheter tips (19.7%), urine (16.7%), ETT (9.1%), and sputum (9.1%) [11], and their study also identified colistin-resistant *P. aeruginosa* isolated from pus and urine specimens, which is similar to our study [11]. In the current study, urine (n = 38) was the most common specimen, followed by pus, the second most frequent specimen (n = 25). Zaki et al. reported the highest number of colistin-resistant isolates from urine (46%), followed by blood (30%) and exudates (24%) [12].

This study evaluated different methods for testing the susceptibility of colistin-resistant Gram-negative bacilli. Currently, BMD is the sole approved method for determining colistin susceptibility patterns [6].

The automated VITEK® 2 Compact system has been documented to be a dependable method for colistin testing [13]. In the current study using the VITEK® 2 Compact automated system, five Gram-negative bacilli isolates were found to be resistant, with MIC values ≥ 4 µg/mL, classified as resistant according to the manufacturer's guidelines. However, compared to the BMD method, the VITEK® 2 Compact was unsuccessful in detecting colistin-resistant *P. aeruginosa *isolates. A total of 92 isolates were tested using the VITEK® 2 Compact and BMD methods, and the findings were analyzed. Among 92 isolates tested, 51 (55.43%) isolates were sensitive, and 41 (44.56%) were found resistant by VITEK® 2. Also, among 92 *P. aeruginosa* isolates tested by BMD, 33 isolates (35.86%) were determined to be susceptible to colistin sulfate (MIC ≤ 0.5 µg/mL), and the rest of 59 (64.13%) isolates were resistant to colistin. We calculated the sensitivity and specificity for the susceptibility category based on the BMD reference standard in all isolates. The highest resistance was shown for the BMD. The differences between these two methods were found to be statistically significant, with the outcome of susceptibility testing by the BMD method, and VITEK® 2 method showing moderate agreement.

In the research conducted by Diana Albertos Torres, 56% of isolates were colistin resistant by the standard BMD testing, and 44% of isolates were susceptible [14]. Tan et al. reported results for colistin susceptibility testing for *Acinetobacter *spp. isolates [15].

VITEK® 2 Compact has been reported as reliable in some studies, but not in others [16,17]. According to the above findings, the VITEK® 2 Compact system can be considered a dependable method for determining colistin susceptibility, though it demonstrates only a moderate degree of reliability [18]. The VITEK® 2 Compact method for colistin susceptibility testing can be trusted for assessing colistin resistance in isolates from genera that generally do not exhibit resistant subpopulations [19]. This research was conducted only on 92 clinical isolates of *P. aeruginosa* that were resistant by BMD in the study period, limiting the generalization of the result. A large-scale, multicenter study is needed in the future for the automation of colistin susceptibility standardization.

## Conclusions

Colistin is now more commonly used to treat life-threatening infections caused by MDR Gram-negative bacteria. In this study, we encountered some isolates that were sensitive on the VITEK® 2 Compact system but were resistant using the BMD method.

The statistical analysis of our study showed a moderate kappa agreement between the susceptibility testing results of the BMD method and the VITEK® 2 Compact method. For routine colistin MIC testing, the VITEK® 2 Compact system provided results that were consistent with the BMD technique; however, resistant subpopulations should be further confirmed using the BMD technique. BMD remains the most sensitive, reliable, and cost-effective approach for colistin susceptibility testing. Its main limitation is that it requires skilled personnel and can be time-consuming. As a result, the VITEK® 2 Compact automated system serves as a viable alternative.
